# Female genital mutilation among children in Ethiopia: A time-to-event analysis of age at circumcision

**DOI:** 10.1371/journal.pone.0317966

**Published:** 2025-01-24

**Authors:** Michael Arthur Ofori, Daniel Biftu Bekalo, David Kwamena Mensah, Niharika Jha

**Affiliations:** 1 School of Public Health, University of Memphis, Memphis, Tennessee, United States of America; 2 Department of Statistics, University of Cape Coast, Cape Coast, Ghana; 3 Department of Mathematics, Pan African University Institute for Basic Sciences Technology and Innovation, Nairobi, Kenya; 4 College of Health and Medical Sciences, Haramaya University, Dire Dawa, Ethiopia; Higher Education Partnership / Erasmus University Rotterdam, ETHIOPIA

## Abstract

Female Genital Mutilation (FGM) has become a global health concern. It is a deeply entrenched harmful practice involving partial or total removal of the external female genitalia for non-medical reasons. To inform effective policymaking and raise awareness about FGM’s health risks, understanding socioeconomic and demographic factors influencing the timing of girls’ circumcision is crucial. This study employed semi-parametric survival models to examine the association between residential status and time-to-circumcision of girls in Ethiopia. The data used in this study was the 2016 Ethiopian Demographic and Health Survey (EDHS) report, which was conducted by the Central Statistical Agency (CSA). The analysis revealed that residential status (rural vs urban, HR = 1.73: 1.35–2.70), religion (Muslim vs Christian, HR = 1.51: 1.38–2.70), mother’s educational level (none vs higher, HR = 5.25: 2.23–12.36 or primary vs higher HR = 4.12: 1.25–9.68), father’s educational level (none vs higher, HR = 1.65: 1.12–2.43), and mother’s age (15–24 years vs 35 +, HR = 1.89: 1.21–2.95 or 24–34 years vs 35 +, HR = 1.55: 1.18–2.02) are significant risk factors for age at circumcision. Mother’s age and family wealth index were time dependent risk factors. Effective interventions to reduce FGM prevalence would need to address multiple aspects simultaneously, including improving access to education (especially for girls and women), targeting both urban and rural areas with awareness campaigns, and considering culturally sensitive approaches that take into account religious and traditional beliefs while promoting the health and rights of girls and women.

## Introduction

Female Genital Mutilation (FGM), a deeply entrenched harmful practice involving partial or total removal of external female genitalia for non-medical reasons, affects millions worldwide. According to the World Health Organization (WHO), the circumcision is any surgery involving the partial or complete removal of the external female genitalia or any other harm to the female genital organs performed for non-medical purposes [1]. The circumcision is often performed on girls from infancy to adolescence in unsanitary conditions by traditional practitioners, risking the girl child to immediate and long-term complications [2].

According to the World Health Organization [3], FGM is practiced in at least 30 countries, primarily in Africa, the Middle East, and Asia. An estimated 200 million girls and women alive today have undergone FGM. UNICEF reported that the practice is most common in the western, eastern, and north-eastern regions of Africa, as well as in some countries in the Middle East and Asia [4]. The United Nations considers FGM a violation of human rights. As of 2023, 51 countries have laws against FGM [5]. However, enforcement remains a challenge in many regions.

FGC can have devastating immediate and long-term consequences for the health and well-being of girls and women. Immediately after the procedure, victims may experience excruciating pain, excessive bleeding, swelling of genital tissues, fever, infections like tetanus, urinary problems, wound healing issues, injury to surrounding genital areas, shock, and even death [1]. However, the complications do not end there. In the long run, girls and women may suffer from urinary problems such as painful urination and urinary tract infections, vaginal issues like discharge, itching, bacterial vaginosis, and other infections, menstrual problems like painful periods and difficulty passing menstrual blood, scar tissue formation and keloids, sexual problems including pain during intercourse and decreased satisfaction, increased risk of childbirth complications such as difficult delivery, excessive bleeding, caesarean section, the need to resuscitate the baby, and newborn deaths, as well as psychological problems like depression, anxiety, post-traumatic stress disorder, and low self-esteem [1].

Based on UNICEF’s March 2024 press release, “over 230 million girls and women alive today have been subjected to female genital mutilation”. Out of this number, nearly 125 million women and girls in Africa and the Middle East have undergone circumcision and other forms of genital mutilation [6]. WHO estimates suggest that globally, millions of women and girls undergo circumcision of which about 2 to 3 million girls are put in danger yearly [7].

East African nations like Somalia (98%), Djibouti (93%), Eritrea (89%), and Ethiopia (74%) have among the highest FGM rates globally [1]. Ethiopia, one African country where this practice remains profoundly rooted in the social norms, is home to approximately half of those affected women [8]. FGM in Ethiopia cuts across most regions and ethnic groups, with a 79.9% national prevalence among girls and women aged 15–49 years Afar (91.6%), Somali (97.3%) regions, and Dire Dawa city (92.3%) exhibit exceptionally high rates [9]. This unsterile practice perpetuates due to tradition and illegality in many areas. FGM imposes substantial public health burdens through increased mortality and lifelong morbidity concerns for affected women and girls [10]. To inform effective policymaking and raise awareness about FGM’s health risks, understanding socioeconomic and demographic factors influencing the timing of girls’ circumcision is crucial. Although many studies have examined these relationships, there are still gaps in our understanding.

This study employed semi-parametric survival models; a statistical method not commonly applied in this context to examine factors associated with time-to-circumcision of children (girls) in Ethiopia. This approach offers new insights into the timing and dynamics of FGM risk. Survival analysis allows you to examine not just if FGM occurs, but when it is most likely to happen in a girl’s life. This method has the power to reveal patterns that previous studies have missed such as age-specific risk factors, critical time windows for intervention as well as how the influence of demographic and SES factors changes over a girl’s lifetime.

## Material and methods

### Data source and study participants

The data for this study was extracted from the 2016 Ethiopian Demographic and Health Survey (EDHS) report, which was conducted by the Central Statistical Agency (CSA) of Ethiopia as part of the global Demographic and Health Surveys (DHS) program. The 2016 EDHS data was collected from January 18, 2016, up to June 27, 2016. The CSA provided a written consent to participant assuring them of confidentiality and anonymity. The authors did not obtain ethical approval since they were not the primary data collectors. The authors had no access to information that could identify individual participants during or after the data collection. The study population comprises 2,615 children (girls) aged 1 to 10 years selected from a total of 15,684 girls in the sampled household clusters.

### Dependent variables

The response or outcome variable is the time-to-circumcision of the girl child, measured in years from birth to the age at which the girl underwent female genital mutilation (FGM). Girls that were circumcised within the time frame were considered event and those that were not circumcised were considered censored.

### Exposure variables

The primary exposure variable was the residential status of the mother (rural or urban).

### Potential confounding variables

Potential risk factors or covariates for FGM include socio-economic, demographic, and environmental factors, such as mother’s education level, religion, household wealth index, exposure to media, employment status, father’s education level, and mother’s age.

### Kaplan-Meier (KM) estimator

The general KM survival probability at a given failure time *t*_(*δ*)_ is defined as the probability of surviving past the previous failure time *t*_(*δ*1)_ as a product of the conditional probability of surviving past time *t*_(*δ*)_. This is written mathematically as;

$$\hat{S}\left(t_{\left(\delta \right)}\right)=\hat{S}\left(t_{\left(\delta -1\right)}\right)*P\left(T>t_{\left(\delta \right)}|T\geq t_{\left(\delta \right)}\right)$$ (1)

Eq (1) in the product limit form can be shown as;

$$\hat{S}\left(t_{\left(\delta -1\right)}\right)=\prod_{i=1}^{\delta -1}\hat{P}r\left(T>t_{i}|T\geq t_{i}\right)$$ (2)

### The Log-Rank Test (LRT)

The log-rank test is used to test the null hypothesis that the survival experience is equal between two or more groups. The log-rank test follows a Chi-square distribution with number of groups less one degrees of freedom (M—1), and it is given by

$$LRT=\frac{\left(O_{i}-E_{i}\right)}{Var\left(O_{i}-E_{i}\right)}$$ (3)

where *Var*(*O*_*i*_ − *E*_*i*_) is the variance. For two groups, the variance is computed as

$$Var\left(O_{i}-E_{i}\right)=\sum_{j}\frac{\left(n_{1}\delta \times n_{2}\delta \right)\left(m_{1}\delta +m_{2}\delta \right)\left(n_{1}\delta +n_{2}\delta -m_{1}\delta -m_{2}\delta \right)}{{\left(n_{1}\delta +n_{2}\delta \right)}^{2}\left(n_{1}\delta +n_{2}\delta -1\right)}$$ (4)

### Cox Proportional Hazard (Cox PH) model

If *X*_1_, *X*_2_, …, *X*_*p*_ are variables that satisfy the proportional hazards assumption, then the general Cox proportional hazard (PH) model is given by

$$h\left(t,X\right)=h_{0}(t)\text{exp}\left\{\beta_{1}X_{1}+\beta_{2}X_{2}+\ldots +\beta_{p}X_{p}\right\},$$ (5)

where *h*_0_(*t*) depends on time but not the *X*_*i*_ while $e^{\sum_{i=1}^{p}\beta_{i}X_{i}}$ depends on the *X*_*i*_ but not time. Cox PH model holds the assumption of proportional hazards signifying that the hazard rate is constant over time. The hazard rate (HR) is given by

$$HR=\frac{\hat{h}\left(t,X^{*}\right)}{\hat{h}\left(t,X\right)}$$ (6)

If Eq 6 is not a constant, we can consider other models like the Stratified Cox (SC) model, Extended SC model, and other time dependent models.

### Stratified Cox (SC) model

Let *X*_1_, *X*_2_, …, *X*_*p*_ be variables that satisfy the proportional hazards assumption and *Z*_1_, *Z*_2_, …, *Z*_*p*_ be a set of variables that do not satisfy the proportional hazards assumption. We define a new variable Z* which is the combination of the various categories of *Z*_*i*_ Let K* also denote the total number of categories of Z* Then the general SC model is given by;

$$h_{g}\left(t,X\right)=h_{0g}(t)\text{exp}\left\{\beta_{1}X_{1}+\beta_{2}X_{2}+\ldots +\beta_{p}X_{p}\right\},$$ (7)

where *g* = 1, 2, …, K* In model (1), the Z* does not appear in the model.

### Extended Cox (EC) model

Let *X*_1_, *X*_2_, …, *X*_*p*_ be time independent variables and *Z*_1_(*t*), *Z*_2_(*t*), …, *Z*_*p*_(*t*) be time dependent variable. A survival model analysis that combines both the time independent and the time dependent variables is the Extended Cox model. The general form of the EC model is given by;

$$h_{g}\left(t,X\right)=h_{0}(t)\text{exp}\left\{\sum_{i=1}^{p}\beta_{i}X_{i}+\sum_{i=1}^{q}\delta_{i}Z_{i}(t)\right\}$$ (8)

### Statistical analyses

The summary statistics were presented using frequency tables. Kaplan-Meier curves was used to evaluate the survival probabilities of time-to-circumcision of the children. The Log-Rank test was employed to compare the survival curves across different levels of residence, religion, wealth index, employment status, and parental educational levels. The goodness of fit methods was used to assess the proportional hazards assumption for appropriate categorical variables. Extended Cox model was developed.

## Results and discussion

In this section, we analyzed the Female Genital Mutilation data and presented the results of our analysis based on the survival model data analysis techniques.

**Fig 1:**
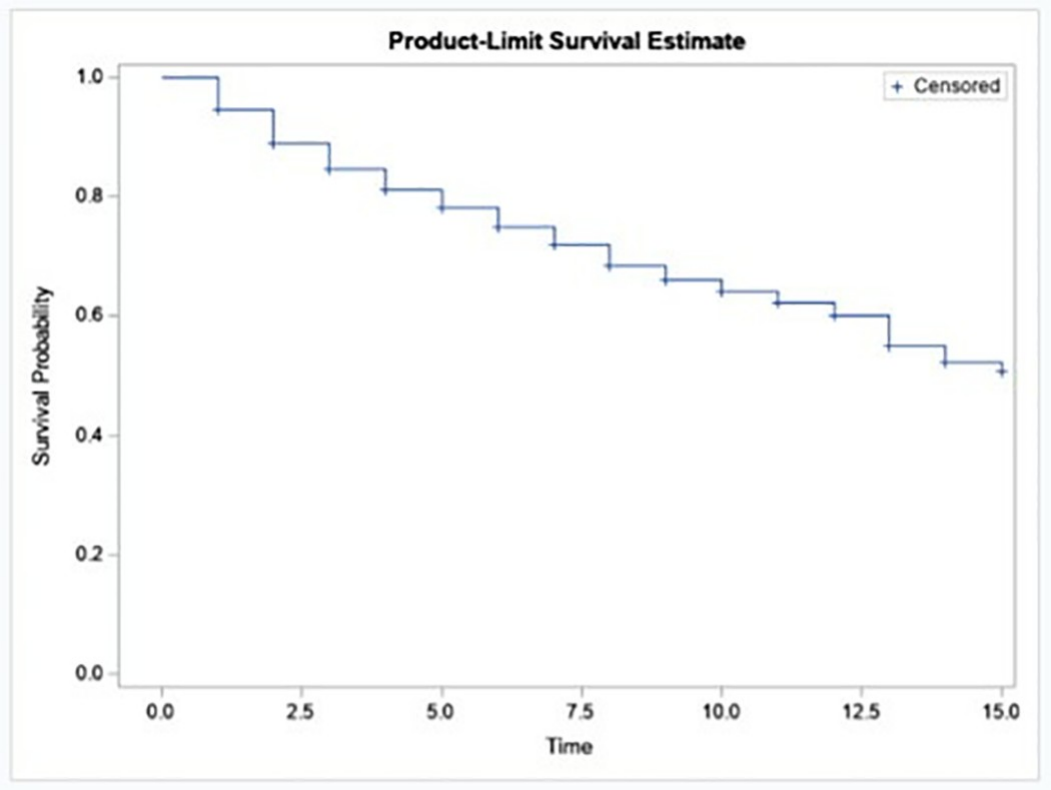
Kaplan Meier plot of survival.

Fig 1 shows the Kaplan-Meier survival plot of time to circumcision of girls in Ethiopia. This is a graphical representation of the survival function over time. It estimates the probability of survival at different time points. Plots of the KM curves show the survival experience of time-to-circumcision of a girl in Fig 1. The survival plot decreases as time progresses, indicating that girls are more likely to get circumcised as they reach a later age.

**Fig 2:**
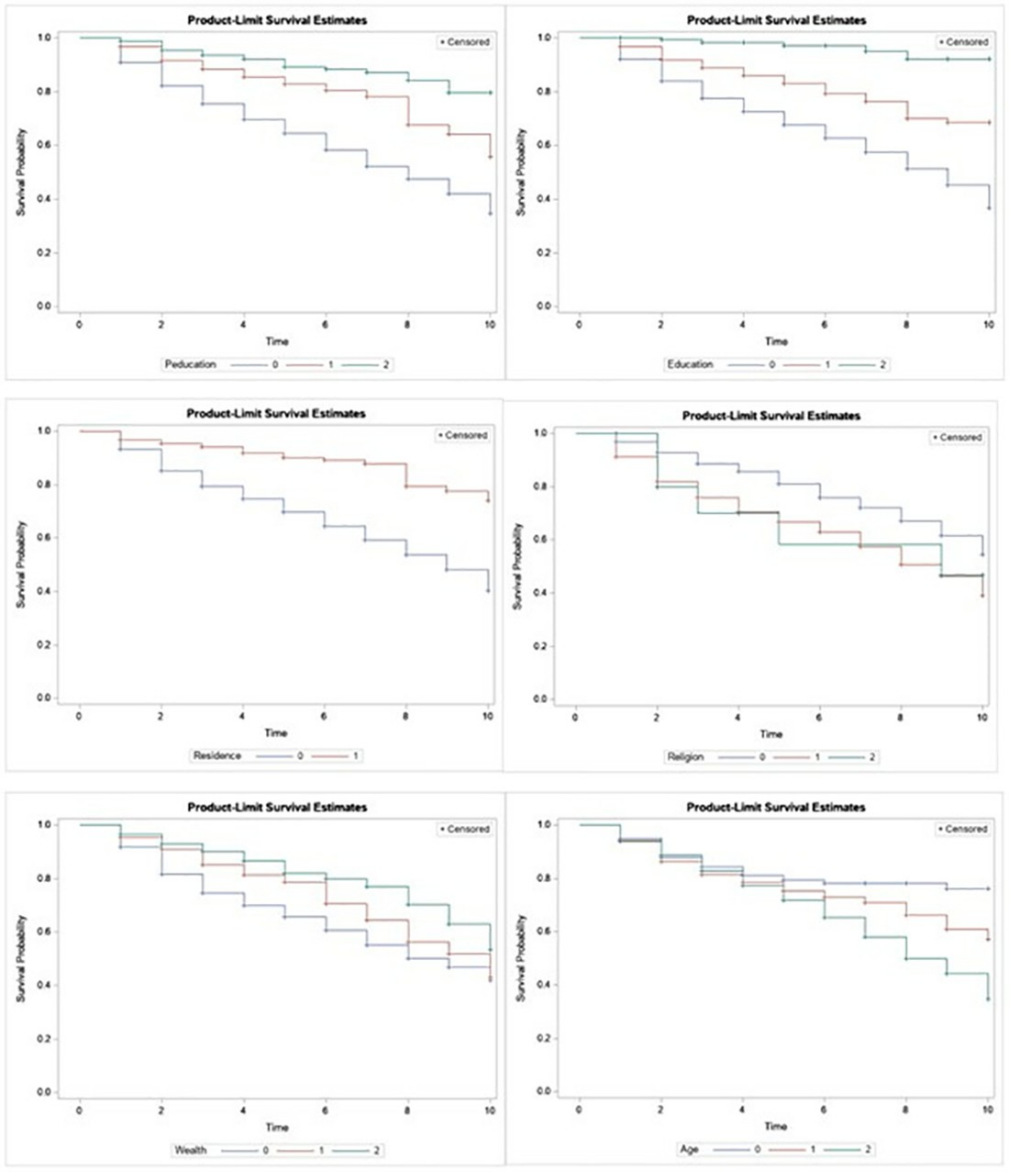
Survival plot of selected variables.

Table 1 presents the results of a Log-rank test, which is a statistical test used to compare the survival distributions of two or more groups. The variables considered are residence, religion, wealth index, Age of mother, Mother’s education, and Father’s education. The p-values indicate that all these variables have a statistically significant association with survival. That is, the time-to-circumcision was different for children living in urban and rural areas, religion (Christians, Muslims, and others), family wealth index (poor, medium, and rich), parents’ educational level (none, primary, secondary/higher). These disparities are visually shown in Fig 2.

**Table 1:** A Log-Rank test of some selected variables.

Variables	Event	Censored	Log-Rank Test		
			Chi-Square	df	P-value
**Residence**			72.88	1	< 0.0001
Rural	567	1510			
Urban	50	488			
**Religion**			62.92	2	< 0.0001
Christian	223	1089			
Muslims	388	896			
Others	6	13			
**Wealth Index**			68.56	2	< 0.0001
Poor	369	860			
Middle	86	311			
Rich	162	827			
**Age of Mother**			18.37	1	0.0001
15–24	56	337			
25–34	243	1006			
35+	318	655			
**Mother’s Education**			110.53	2	< 0.0001
None	519	1201			
Primary	92	550			
Secondary or higher	6	247			
**Father’s Education**			135.81	2	< 0.0001
None	456	912			
Primary	125	671			
Secondary or higher	36	415			

The survival plots in Fig 2 illustrate the time-to-circumcision of girls based on their mothers’ place of residence, age, wealth index, education, father’s education and religion. The plot indicates that the risk of circumcision is similar for girls born to mothers in rural or urban areas, but differences become visible in the middle and toward the end of the curves. From the middle to the end, the survival plot for children born to rural mothers is lower than that for girls born to urban mothers, suggesting a higher risk of circumcision for girls born to rural mothers. The plot also shows that girls born to mothers aged 35–49 have the highest risk of circumcision compared to other age groups. Additionally, girls born to mothers with a poor wealth index are at higher risk of circumcision compared to those born to mothers with middle or rich wealth indexes. Furthermore, girls born to mothers practicing the Muslim faith or other religions are at higher risk of circumcision compared to those born to Christian mothers. The plot also indicates that initially, the risk of circumcision is similar for all groups, but differences become visible in the middle of the curves. At this point, the survival plot for girls whose mothers have no education is lower than that for girls whose mothers have primary, secondary, or higher education, indicating a higher risk of circumcision for girls whose mothers have no education. The plot also shows that girls whose fathers have no education are at higher risk of circumcision compared to those whose fathers have completed primary school or higher education.

**Fig 3:**
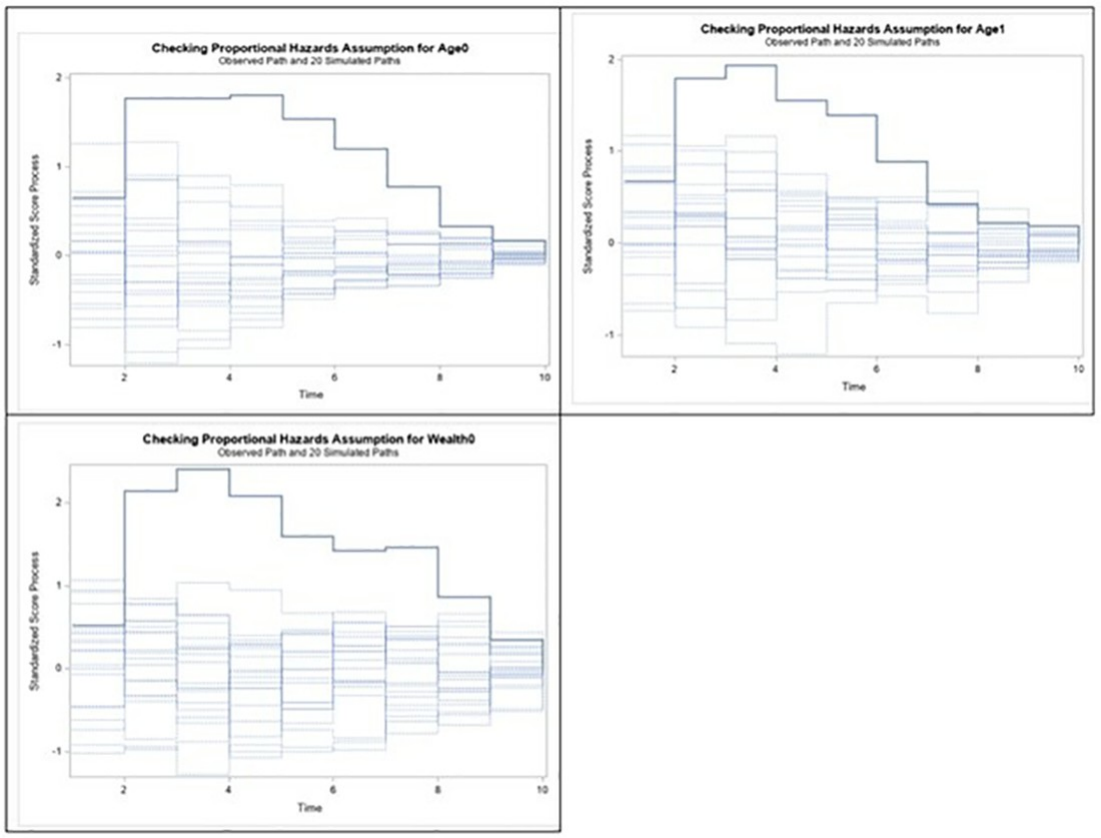
Test of proportional hazard assumption for selected variables.

The test of proportional assumption revealed that the test holds for all the variables except wealth index of the family and the mother’s age as shown in Fig 3. This suggests that it will not be appropriate to run a Cox proportional model with wealth index together with the other variables. As a result, we opted for the extended Cox Model as shown in Table 2.

**Table 2:** The Extended Cox model.

Parameter	Estimate	Error	Chi-Square	Pr>ChiSq	HR	95% CI
Residence (Ref = Urban)						
Rural	0.6562	0.1714	14.6660	0.0001	1.93	1.38–2.70
Religion (Ref = Christian)						
Islam	0.4137	0.0894	21.3895	< 0.0001	1.51	1.27–1.80
Others	0.4418	0.4181	1.1163	0.2907	1.56	0.69–3.53
Mother’s Age (Ref = 35+)						
15–24	0.6358	0.1806	7.7926	0.0052	1.89	1.21–2.95
25–34	0.4351	0.1387	9.8351	0.0017	1.55	1.18–2.03
Wealth Index (Ref = Rich)						
Poor	0.3649	0.1806	4.0810	0.0434	1.44	1.01–2.05
Middle	0.0147	0.1634	0.0081	0.9282	1.02	0.74–1.40
Mother’s Education (Ref = Secondary or higher)						
None	1.6574	0.4371	14.3746	0.0001	5.25	2.23–12.36
Primary	1.4160	0.4357	10.5586	0.0012	4.12	1.75–9.68
Father’s Education (Ref = Secondary or higher)						
None	0.5018	0.1965	6.6212	0.0107	1.65	1.12–2.43
Primary	-0.0139	0.2014	0.0048	0.9449	0.99	0.67–1.46
Wealth index_t	0.0504	0.0187	7.2824	0.0070	1.05	1.01–1.09
Mother’s Age_t	0.1735	0.0314	30.5199	<0.0001	1.065	1.12–1.27

Table 2 presents the results of the Extended Cox model, examining various socio-demographic factors and their influence on the hazard ratio (HR) for age at circumcision of children in Ethiopia. The table includes parameter estimates, standard errors, Chi-square statistics, p-values, hazard ratios, and their 95% confidence intervals (CI) for different covariates. Key factors analyzed include residence, religion, mother’s age, wealth index, and education levels of both mother and father.

The findings indicate that children residing in rural areas have a significantly higher risk (HR = 1.93, 95% CI: 1.38–2.70, p-value = 0.0001) of been circumcised as compared to those in urban areas. Similarly, those whose parents are Muslims have a higher risk (HR = 1.51, 95% CI: 1.27–1.80, p-value < 0.0001) of been circumcised compared to those whose parents are Christians, while other religions did not show significant results. Younger mothers (aged 15–24 years) and those aged 25–34 years have higher risks (HR = 1.89 and 1.55, respectively) of allowing their girl-child to be circumcised than those aged 35 years and above. Lower wealth indices (poor vs. rich) and lower levels of parental education are major contributing factors to the age at circumcision of children in Ethiopia. Notably, children born to mothers with no education (HR = 5.25, p-value = 0.0001) or only primary education (HR = 4.12, p-value = 0.0012) face significantly higher risks. Also, children born to fathers with no education (HR = 1.65, p-value = 0.0107) face significantly higher risks of been circumcised, emphasizing the profound impact of educational attainment on health outcomes. Additionally, changes in the wealth index and mother’s age considerably affect the hazard ratios, indicating dynamic influences over time.

## Discussion

The main goal of the study was modelling the determinants of female genital mutilation among children (young girls aged ten years and below) in Ethiopia using extended Cox model. This method shows how the risk of FGM changes over time and in relation to various factors, providing a more nuanced understanding than static models. This temporal dimension can reveal critical periods of risk. Looking at the problem of FGM from this lens can handle censored data, accounting for girls who haven’t undergone FGM by the end of the study period but may still be at risk. The insights from survival analysis can inform more targeted and timely interventions. Lastly, this study did not only contribute new knowledge about FGM in Ethiopia but also demonstrate the value of survival analysis in studying other similar harmful practices.

The residential status was used as the exposure variable in modelling the time-to-event analysis of age at circumcision of girls in Ethiopian using 2016 EDHS data. The Log-rank test revealed that residence, religion, wealth index, and mother’s education have a statistically significant association with survival. The extended Cox model results suggested that place of residences was significant risk factor for age at circumcision of girls in Ethiopia. This shows that girls who lived in urban areas had longer survival with respect to age at circumcision than girls who reside in rural areas. It showed that the risk of age at circumcision for girls who lived in rural area was more than twice those who live in the urban. A similar study that was conducted in Ethiopia by [11] revealed that the rural girls were more vulnerable towards circumcision than urban girls. Another study that was conducted in Somalia by [12] revealed that daughters of women residing in an urban area were 70% less likely to be at risk of FGM. [13] found the prevalence of FGM of girls younger than 15 years to be higher among those living in the rural areas compared to those in urban areas (AOR 6.74). Other studies [14, 15] also reported that girls living in urban areas have a reduced risk of FGM. Although [16] found no significant association between residence and FGM among Kenyan girls (aged 0–14 years) yet we cannot talk about the significant risk factors of FGM without mentioning residential status of the people involved. Most people in rural areas are deep rooted in tradition and lack so much knowledge as far as modernity is concerned.

Generally, FGM tends to be more common in rural areas than in urban areas. This is often attributed to stronger adherence to harmful practices and less exposure to education and awareness campaigns in rural settings. Urban residents typically have greater access to education, healthcare, and media, which can increase awareness about the harmful effects of FGM and challenge traditional beliefs supporting the practice but that is not the case in the rural areas. Also, in rural communities, social cohesion is often stronger, which can lead to increased pressure to conform to harmful practices like FGM. Urban areas may offer more anonymity and less community pressure. Religion was also found to be a risk factor for determining age at circumcision of FGM. It showed that girls whose parents are Muslims had a hazard of 1.5 times higher than girls whose parents were Christians. A similar study conducted by [17] found that girls whose mothers are Muslims are more vulnerable towards circumcision. Other studies [13, 18–20] have also found religion to be a significant risk factor of FGM. Religion plays a vital part in our lives as people, and we cannot do away without it yet that should not be used as a yardstick to abuse innocent people. The various religions must be made to understand the side effect of this practice on the future of this little girls.

The findings of this study also revealed that the educational level of parents had a significant effect on the survival of age at circumcision. Girls whose mothers had no education had risk that is five times higher than girls whose mothers had secondary or higher education. Also, girls whose mothers had primary education had risk that is four times higher than those girls whose parent had higher education. Education indeed has a significant impact on health outcomes and this is evident. This result is consistent with the study conducted in Burkina Faso by [21] which revealed that the prevalence of girls’ circumcision is high for those daughters whom their mothers are illiterate. [22] found that women with at least secondary education are less likely to genitally mutilate their daughters (AOR = 0.248). Also, [15] reported that higher maternal education is among the factors that reduces the odds of FGM. Paternal education is also a key factor as far as FGM is concerned. Our result showed that girls whose fathers had no education were at a higher risk of been circumcised (1.65) compared to those whose fathers had secondary or higher education. Comparatively, the paternal education was not as severe as that of the maternal education.

Although education alone cannot be the only solution to FGM, but it is a very important factor as far as FGM is concerned. While paternal education plays a role in reducing FGM prevalence, its impact is often less direct as compared to mothers. Fathers with higher education levels may be more likely to support their wives’ decisions to abandon the practice or may be more open to changing cultural norms. Mother’s educational level is very important in this discussion because in many societies where FGM is practiced, mothers are often the primary caregivers and have more direct influence over decisions related to their daughters’ health and cultural practices. Also, women who have undergone FGM themselves are more likely to support the practice for their daughters. Higher maternal education can break this cycle by increasing awareness of the health risks and human rights implications. Likewise, educated mothers may have more autonomy and decision-making power within the family unit, allowing them to oppose FGM even if it goes against community norms. Lastly, maternal education often correlates with increased health literacy, making mothers more aware of the medical complications associated with FGM.

The result of the study showed that girls whose family wealth index is considered poor had a higher risk than those whose family wealth status is considered rich. A study by [23] found the family wealth as significant risk factors of FGM. According to Geremew et al. (2021), a better family wealth can reduce the risk of FGM. We also found that younger mothers (15–24 years and 25–34 years) had a higher risk of circumcising their children as compared to older mothers (35 years and above) with hazard risk of 1.85 and 1.55 respectively. A mother’s age was found to be time dependent as aged, the risk of allowing their children to be circumcised reduces. This confirms the assertion of [15] that older maternal age decreases the odds of FGM.

There is likely a strong correlation between education levels and residential status. Urban areas typically offer better access to education, which may explain why both urban residence and higher parental education are protective factors against early FGM. Likewise, educational levels may vary among different religious groups, potentially influencing the observed differences in FGM risk between Muslims and Christians. Mother’s age, education, and residential status are linked in some way as far as these results are concerned. Younger mothers are more likely to have lower education levels and live in rural areas, which could compound the risk factors for early FGM. Wealthier families are more likely to live in urban areas and have higher education levels, which may contribute to the protective effect against early FGM. These interrelationships highlight the complex, multifaceted nature of the factors influencing FGM practices. Effective interventions to reduce FGM prevalence would need to address multiple aspects simultaneously, including improving access to education (especially for girls and women), targeting both urban and rural areas with awareness campaigns, and considering culturally sensitive approaches that take into account religious and traditional beliefs while promoting the health and rights of girls and women.

## Conclusions

The study reveals a significant difference in survival experience among the various categories of variables that were considered. Residential status, religion, wealth index, mother and father’s educational level, and mother’s age are significant risk factors for FGM. Mother’s age and family wealth index were found to be time dependent. The odds of a girl child whose mother has no education been circumcised is 5.3 times the one whose mother has a higher education.

## Supporting information

S1 Data (XLSX)
